# Prevalence and Genetic Characteristics of *Staphylococcus aureus* CC398 Isolates From Invasive Infections in Spanish Hospitals, Focusing on the Livestock-Independent CC398-MSSA Clade

**DOI:** 10.3389/fmicb.2021.623108

**Published:** 2021-02-09

**Authors:** Olouwafemi Mistourath Mama, Carmen Aspiroz, Laura Ruiz-Ripa, Sara Ceballos, Maria Iñiguez-Barrio, Emilia Cercenado, José Manuel Azcona, Lorena López-Cerero, Cristina Seral, Ana Isabel López-Calleja, Alba Belles-Belles, Pilar Berdonces, María Siller, Myriam Zarazaga, Carmen Torres, the Study Group of clinical S. aureus CC398

**Affiliations:** ^1^Área Bioquímica y Biología Molecular, Universidad de La Rioja, Logroño, Spain; ^2^Servicio de Microbiología, Hospital Royo Villanova, Zaragoza, Spain; ^3^Servicio de Microbiología, Hospital Universitario Gregorio Marañón, Madrid, Spain; ^4^Servicio de Microbiología, Hospital San Pedro, Logroño, Spain; ^5^Servicio de Microbiología, Hospital Universitario Virgen Macarena, Sevilla, Spain; ^6^Servicio de Microbiología, Hospital Clínico Universitario Lozano Blesa, Zaragoza, Spain; ^7^Servicio de Microbiología, Hospital Universitario Miguel Servet/IIS Aragón, Zaragoza, Spain; ^8^Servicio de Microbiología, Hospital Universitario Arnau de Vilanova, Lérida, Spain; ^9^Servicio de Microbiología, Hospital Galdakao, Galdakao, Spain; ^10^Servicio de Microbiología, Hospital Universitario Marqués de Valdecilla, Santander, Spain

**Keywords:** MSSA, LA-MRSA, CC398, bacteremia, t571, t1451, *erm*(T), Spain

## Abstract

**Background:**

Livestock-associated (LA)-CC398-MRSA is closely related to pigs, being unfrequently detected in human invasive infections. CC398-MSSA is emerging in human invasive infections in some countries, but genetic and epidemiological characteristics are still scarcely reported.

**Objectives:**

To determine the prevalence of *Staphylococcus aureus* (SA) CC398, both MRSA and MSSA, among blood cultures SA isolates recovered in Spanish hospitals located in regions with different pig-farming densities (PD) and characterize the recovered isolates.

**Methods:**

One thousand twenty-two SA isolates (761 MSSA, 261 MRSA) recovered from blood cultures during 6–12 months in 17 Spanish hospitals (2018–2019) were studied. CC398 lineage identification, detection of *spa*-types, and antibiotic resistance, virulence and human immune evasion cluster (IEC) genes were analyzed by PCR/sequencing.

**Results:**

Forty-four CC398-MSSA isolates (4.3% of SA; 5.8% of MSSA) and 10 CC398-MRSA isolates (1% of SA; 3.8% of MRSA) were detected. Eleven *spa*-types were found among the CC398-MSSA isolates with t571 and t1451 the most frequent *spa*-types detected (75%). Most of CC398-MSSA isolates were Immune-Evasion-Cluster (IEC)-positive (88.6%), tetracycline-susceptible (95.5%) and erythromycin/clindamycin^–inducible^-resistant/*erm*(T)-positive (75%). No statistical significance was detected when the CC398-MSSA/MSSA rate was correlated to PD (pigs/km^2^) (*p* = 0.108). On the contrary, CC398-MRSA isolates were all IEC-negative, predominately *spa*-t011 (70%), and the CC398-MRSA/MRSA rate was significantly associated to PD (*p* < 0.005).

**Conclusion:**

CC398-MSSA is an emerging clade in invasive infections in Spanish hospitals. CC398-MRSA (mostly t011) and CC398-MSSA (mostly t571 and t1451) show important differences, possibly suggesting divergent steps in host-adaptation evolutionary processes. While CC398-MRSA is livestock-associated (lacking IEC-system), CC398-MSSA seems to be mostly livestock-independent, carrying human-adaptation markers.

## Introduction

Livestock-associated (LA) methicillin-resistant *S. aureus* (MRSA) of clonal complex (CC) 398 has gained much attention during the past decade because, apart from colonizing farm-animals, it has become a frequent pathogen in humans mainly -but not always- in contact with livestock (Lozano et al., 2012; Benito et al., 2014; Becker et al., 2017; Murra et al., 2019). More recently, methicillin-susceptible *S. aureus* (MSSA) of lineage CC398 has been increasingly reported as a cause of invasive infections in patients without livestock contact in different European countries, mainly in France, but also in Portugal or Belgium (Valentin-Domelier et al., 2011; Vandendriessche et al., 2011; Tavares et al., 2014; Bouiller et al., 2016; Bonnet et al., 2018; Sauget et al., 2019; Bouiller et al., 2020). Out of Europe, CC398-MSSA related human infections have been detected in other countries, as EEUU, and increasingly found in China (Mediavilla et al., 2012; Uhlemann et al., 2013; He et al., 2013; Li et al., 2019; Bouiller et al., 2020). CC398-MSSA isolates are easily transmissible among humans and showed a genetic background that is well adapted to the human host (Uhlemann et al., 2012).

Based on whole genome sequencing analysis, LA-CC398-MRSA has evolved from an ancestor human-adapted (HA) methicillin susceptible *S. aureus* (MSSA) CC398 (Price et al., 2012). This CC398-MSSA would have acquired methicillin and tetracycline resistance and lost the prophage ΦSa3, that carries the immune evasion cluster (IEC) genes; IEC is a set of genes which protects *S. aureus* against the human immune system (Van Wamel et al., 2006). The gene *scn* (which encodes the staphylococcal complement inhibitor) is present in all types of IEC and is therefore considered a marker of this cluster (Zarazaga et al., 2018). A subpopulation of CC398-MSSA carrying the IEC system seems to be emerging in human infections, as previously indicated, in patients without livestock exposure, that could have also evolved from the HA-MSSA ancestor (Bouiller et al., 2020). Nevertheless, still there are scarce data related to the dissemination of this CC398-MSSA subpopulation at global level.

As practically all LA-CC398-MRSA show tetracycline resistance (TET^R^), this is considered a marker for LA-CC398-MRSA detection among epidemiological or clinical strains (Lozano et al., 2012; Benito et al., 2014; Ceballos et al., 2019). Based on this observation, a recent multicenter study performed by our research group, established a strong positive correlation between LA-CC398-MRSA prevalence in Spanish hospitals and the Pig-farming Density (**PD**) of surrounding regions (Ceballos et al., 2019). This study found a global prevalence of CC398-MRSA/MRSA of 3.8%. A recent study focused on blood culture isolates in one Spanish hospital revealed a CC398-MSSA prevalence of 5.2% of *S. aureus* and 8% of MSSA, with absence of CC398-MRSA (Mama et al., 2020b).

For the all above and considering that *S. aureus* represents one of the most common causes of bloodstream infections (Bonnet et al., 2018), this study aimed to determine the prevalence of CC398 among blood culture *S. aureus* isolates from 17 Spanish hospitals located in regions with different PD. We also intend to give more information about CC398-MSSA molecular and epidemiological characteristics.

## Materials and Methods

### Strains Collection

A total of 1,022 *S. aureus* isolates (1 isolate/patient) were collected from blood cultures in 17 Spanish hospitals during 2018–19 (12 hospitals: 12 months period; 5 hospitals: 6–9 months period) (Table 1). In this collection, 761 isolates were MSSA and 261 were MRSA (25 of these isolates were MRSA-TET^R^, representing 10% of total MRSA). All 761 MSSA and the 25 MRSA-TET^R^ isolates were included in this study.

**TABLE 1:** Prevalence of MSSA, CC398-MSSA, and LA-CC398-MRSA in blood cultures from 17 hospitals in Spain, located in regions with different pig farming density.

Number of strains									Rate (%)					
Hospital code ^a^	Regions	Pig density (pigs/km^2^)	Isolation time (in months)	*S. aureus*	MSSA	MRSA-TetR	CC398- MSSA	CC398- MRSA	MSSA/- *S. aureus*	CC398-MSSA/ *S. aureus*	CC398- MSSA/MSSA	MRSA/- *S. aureus*	CC398-MRSA/ *S. aureus*	CC398-MRSA/MRSA
H1-HUAV	Lérida	358.4	12^c^	79	57	5	6	5	72.2	7.6	10.5	27.8	6.3	22.7
H2-HSJ	Huesca	258.8	6^c^	11	7	1	2	1	63.6	18.2	28.6	36.4	9.1	25
H3-HB	Huesca	258.8	12^c^	10	8	1	0	0	80	0	0	20	0	0
H4-HUV	Barcelona	256.0	6^c^	17	12	2	1	2	70.6	5.9	8.3	29.4	11.8	40
H5-HUMS	Zaragoza	166.8	12^b^	104	77	1	6	1	74.0	5.8	7.8	26.0	1.0	3.7
H6-HULB	Zaragoza	166.8	12^b^	81	63	5	3	1	77.8	3.7	4.8	22.2	1.2	5.6
H7-HRV	Zaragoza	166.8	12^b^	38	18	1	2	0	47.4	5.3	11.1	52.6	0	0
Hospitals in VHPD regions****				340	242	16	20	10	71.17	5.9	8.3	28.83	2.9	10.2
H8-CHN	Navarra	58.8	12^b^	56	41	2	3	0	73.2	5.4	7.3	26.8	0	0
H9-CUN	Navarra	58.8	12^b^	11	10	0	0	0	90.9	0	0	9.1	0	0
Hospitals in HPD regions***				67	51	2	3	0	76.1	4.5	5.9	23.9	0	0
H10-HUVM	Sevilla	47.8	12^b^	109	90	2	6	0	82.6	5.5	6.6	17.4	0	0
H11-HUB	Burgos	33.3	9^c^	48	32	1	1	0	66.7	2.1	3.1	33.3	0	0
H12-HSP	La Rioja	24.3	12^b^	71	50	0	2	0	70.4	2.8	4.0	29.6	0	0
Hospitals in MPD regions**				228	172	3	9	0	75.4	3.9	5.2	24.6	0	0
H13-HG	Vizcaya	5.0	12^b^	48	35	1	5	0	72.9	10.4	14.3	27.1	0	0
H14-HUA	Álava	5.0	9^c^	49	41	1	2	0	83.7	4.1	4.9	16.3	0	0
H15-HUD	Guipúzcoa	5.0	6^c^	53	45	0	0	0	84.9	0	0	15.1	0	0
H16-HUGM	Madrid	2.2	12^b^	143	108	1	3	0	75.5	2.1	2.8	24.5	0	0
H17-HUMV	Cantabria	0.3	12^b^	94	67	1	2	0	71.3	2.1	3.0	28.7	0	0
Hospitals in LPD regions*				387	296	4	12	0	76.5	3.1	4.05	23.5	0	0
Total of 17 hospitals				1,022	761	25	44	10	74.5	4.3	5.8	25.5	1	3.8

^*a*^Code for the hospitals (H.). The hospitals have been designated from H1-H17 followed by the name of the hospital: HUAV, H. Universitario Arnau Vilanova; HSJ, H. San Jorge; HB, H. Barbastro; HUV, H. Universitari de Vic; HUMS, H. Universitario Miguel Servet; HULB, H. Universitario Lozano Blesa; HRV, H. Royo Villanova; CHN, Complejo Hospitalario de Navarra; CUN, Clínica Universitaria de Navarra; HUVM, H. Universitario Virgen Macarena; HUB, H. Universitario de Burgos; HSP, H. San Pedro; HG, H. de Galdakao; HUA, H. Universitario de Álava; HUD, H. Universitario de Donostia; HUGM, H. Universitario Gregorio Marañon; HUMV, H. Marques Valdecilla.* Hospitals located in a low pig density (LPD) area: 0–10 pigs/km^2^; ** Hospitals located in a medium pig density (MPD) area: 11–50 pigs/km^2^; *** Hospitals located in high pig density (HPD) area: 51–150 pigs/km^2^; **** Hospitals located in very high pig density (VHPD) area: >150 pigs/km^2^. Data were taken from the report of the Ministerio de Agricultura, Pesca y Alimentación, on Pigs in 2018, Spain.^*b*^Isolation Time. 12 months: January–December 2018.^*c*^Isolation Time. 6–12 months of 2019.

**FIGURE 1:**
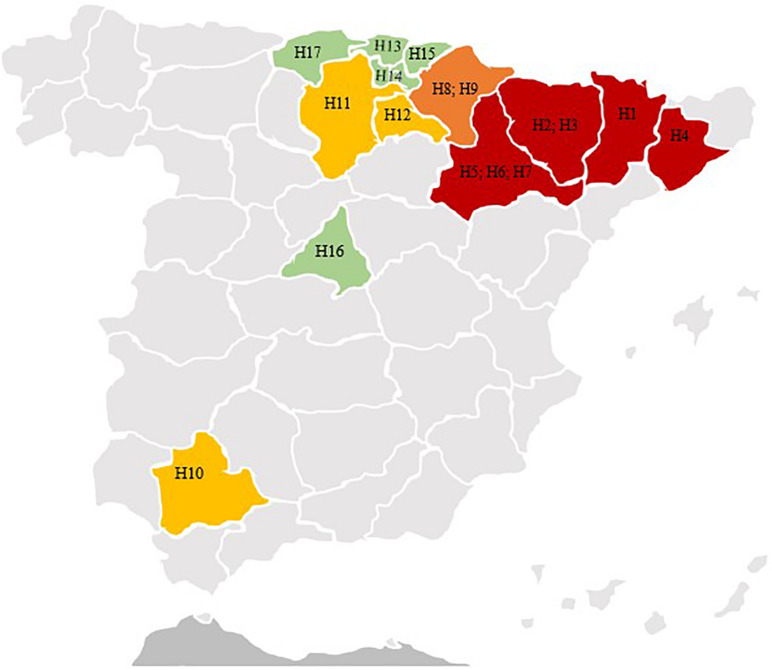
Map of Spain, highlighting the different regions in which are located the hospitals included in this study and the pig density of the regions. Code for hospitals (H). H1, H. Universitario Arnau Vilanova; H2, H. San Jorge; H3, H. Barbastro; H4, H. Universitari de Vic; H5, H. Universitario Miguel Servet; H6, H. Universitario Lozano Blesa; H7, H. Royo Villanova; H8, Complejo Hospitalario de Navarra; H9, Clínica Universitaria de Navarra; H10, H. Universitario Virgen Macarena; H11, H. Universitario de Burgos; H12, H. San Pedro; H13, H. de Galdakao; H14, H. Universitario de Álava; H15, H. Universitario de Donostia; H16, H. Universitario Gregorio Marañón; H17, H. Universitario Marqués de Valdecilla. ■ Regions with low pig density (0–10 pigs/km^2^): Bizcaia (H13); Araba (H14); Gipuzcoa (H15); Madrid (H16); Cantabria (H17). ■ Regions with medium pig density (11–50 pigs/km^2^): Sevilla (H10); Burgos (H11); La Rioja (H12). ■ Regions with high pig density (51–150 pigs/km^2^): Navarra (H8; H9). ■ Regions with very high pig density (>150 pigs/km^2^): Lleida (H1); Huesca (H2; H3); Barcelona (H4); Zaragoza (H5; H6; H7).

The participating hospitals in this multicenter study were located in regions with different PDs (Figure 1), according to data previously published (Ministerio de Agricultura, Pesca y Alimentación, 2018), and they were classified as follows: Low-PD (LPD): 0–10 pigs/km^2^ (5 hospitals); Medium-PD (MPD): 10–50 pigs/km^2^ (3 hospitals); High-PD (HPD): 50–150 pigs/km^2^ (2 hospitals); Very-High-PD (VHPD): >150 pigs/km^2^ (7 hospitals) (Table 1 and Figure 1).

In this study we have analyzed the *S. aureus* isolates obtained in the routine work of the microbiology laboratories as part of the diagnostic process, and no clinical data of patients was used.

The resistance phenotype to eighteen antimicrobial agents was performed using automatic methods and/or disk diffusion tests. Breakpoints were considered according to the European Committee on Antimicrobial Susceptibility Testing and/or the Clinical and Laboratory Standards Institute, depending on hospitals.

### Molecular Characterization

All 761 MSSA and 25 MRSA-TET^R^ isolates included in the study were subjected to specific PCR screening for CC398 lineage (Stegger et al., 2011). *sp*a-typing was carried-out by PCR/sequencing for all CC398-MSSA and for the 25 MRSA-TET^R^ isolates (Benito et al., 2014). Additional characterization was performed by PCR in these isolates as previously described (Benito et al., 2014): (1) antibiotic resistance gene detection according to their antibiotic resistance phenotype; (2) *scn* gene detection and IEC-typing for *scn*-positive strains; and (3) *eta*, *etb*, *tst*, and *lukF/S-PV* gene screening.

### Statistical Analysis

Spearman correlations between pig density (pigs/km^2^) and proportions of data of interest were studied. A *p* < 0.05 was considered statistically significant showing a relationship between the selected variables. These statistical analyses were performed using the RStudio program (version 1.2.5042).

## Results

### Prevalence of CC398 Isolates Among MSSA and MRSA-TET^R^ Isolates

MRSA represented 25.5% of total *S. aureus* isolates recovered from blood culture samples in the studied period (range: 9.1–52.6%). The lineage CC398 was detected in this multicenter study in 5.3% of total *S. aureus* isolates (including both MRSA and MSSA) (Table 1).

The CC398-MSSA isolates detected (*n* = 44) represented 4.3% of total *S. aureus* and 5.8% of MSSA invasive isolates (Table 1). The distribution among the hospitals was heterogenous in our study, with slight differences observed in the prevalence of CC398-MSSA/MSSA when hospitals of VHPD, HPD, MPD, and LPD regions were compared; nevertheless, no statistical significance was detected when the CC398-MSSA/MSSA rate was correlated to PD (*p* = 0.108).

Ten out of the 25 MRSA-TET^R^ isolates (40%) were ascribed to CC398 lineage, representing 3.8% of total MRSA and 1% of all *S. aureus* invasive isolates. All CC398-MRSA isolates were recovered from five hospitals located in regions with VHPD (Table 1). A significant correlation was found between CC398-MRSA/MRSA rate and the PD data (*p* = 0.0023).

### Genetic Characterization of CC398-MSSA Isolates

Eleven different *spa*-types were detected among the 44 CC398-MSSA isolates, being predominantly t571 (*n* = 19; 43.2%) and t1451 (*n* = 14; 31.8%). The other *spa*-types detected were as follows: t011 (*n* = 2), t4030 (*n* = 2), t7880, t899, t034, t1255, and t7160 (*n* = 1, each one); two novel *spa*-types were also found (Table 2).

**TABLE 2:** Characteristics of the 44 CC398-MSSA isolates and the 14 CC398-MRSA and CC1-MRSA isolates recovered from blood cultures from 17 hospitals in Spain.

			Antimicrobial resistance				
	*spa*-type (*n*° isolates)	Hospital^a^ (*n*° isolates)	Phenotype^b^ (*n*° isolates)	Genotype (*n*° isolates)	Virulence genes (*n*° isolates)	*Scn* (*n*° isolates)	IEC-type (*n*° isolates)
CC398-MSSA *n* = 44	t571 (19)	HG (4), HUGM (2), HUMS (2), HSP (2), HUVM (2), HUAV (3), HUA (1), CHN (1), HSJ (1), HUV (1)	ERY, CLI^c^ (12)	*erm*(T) (10)	*eta* (1)	+ (10)	C (7), B (3)
				*erm*(T), *erm*(A), *msr*(A) (1)	–	+	C
				*erm*(T), *erm*(C), *lnu*(A), *vga*(A) (1)	–	+	B
			ERY, CLI^c^, PEN (3)	*erm*(T), *erm*(A), *erm*(C), *vga*(A), *blaZ* (1)	–	+	C
				*erm*(T), *erm*(A), *msr*(A), *blaZ* (1)	–	–	–
				*erm*(T), *lnu*(A), *blaZ* (1)	–	+	B
			PEN (2)	*blaZ* (2)	–	+	B (2)
			Susceptible (2)	–	–	+ (2)	B (2)
	t1451 (14)	HUMS (3), HULB (2), HUVM (2), HRV (1), HUMV (1), CHN (1), HG (1), HUGM (1), HUB (1), HUA (1)	ERY, CLI^c^, PEN (7)	*erm*(T), *blaZ* (2)	–	+ (2)	C (2)
				*erm*(T), *erm*(A), *blaZ* (2)	+	+ (2)	C (2)
				*erm*(T), *erm*(A), *msr*(A), *blaZ* (1)	–	+	C
				*erm*(T), *erm*(A), *msr*(A), *lnu*(A) *blaZ* (1)	*eta*	+	B
				*erm*(T), *msr*(A), *blaZ* (1)	–	+	C
			ERY, CLI^c^ (5)	*erm*(T) (3)	*eta* (1)	+ (3)	C (3)
				*erm*(T), *erm*(A), *msr*(A) (1)	–	+	B
				*erm*(T), *erm*(A), *lnu*(A) (1)	–	+	C
			ERY, CLI^c^, PEN, TOB (1)	*erm*(T), *erm*(C), *msr*(A), *blaZ, ant(4’)–la*	–	+	B
			ERY, CLI**^c^**, PEN, GEN, TOB (1)	*erm*(T), *blaZ, aac(6’)–Ie–aph(2”)–Ia*	–	–	–
	t011 (2)	HUAV (1)	PEN (1)	*blaZ*	–	–	–
		HUMV (1)	Susceptible (1)	–	–	–	–
	t4030 (2)	HRV (1)	ERY, CLI**^c^** (1)	*erm*(T), *erm*(A)	–	+	C
		CHN (1)	ERI–I^d^ (1)	*erm*(T)	–	+	C
	t899 (1)	HULB	PEN, TET	*blaZ*, *tet*(M)	–	+	B
	t034 (1)	HUMS	ERY, CLI^c^, TET	*erm*(B), *msr*(A), *tet*(K)	*eta*	–	
	t1255 (1)	HUVM	Susceptible	–	–	+	E
	t7880 (1)	HUVM	ERY, CLI^c^	*erm*(T), *erm*(A)	–	+	C
	t7160 (1)	HUAV (1)	Susceptible	–	–	+	E
	New (1)	HUAV (1)	PEN (1)	*blaZ*	–	+	C
	New (1)	HSJ (1)	ERY, CLI^c^, PEN (1)	*erm*(T), *lnu*(A) *blaZ* (1)	–	+	B
CC398-MRSA *n* = 10	t011 (7)	HULB (1), HUMS (1), HUAV (3), HSJ (1), HUV (1)	FOX, PEN, TET, ERI, CLI, CIP (2)	*mecA*, *tet*(M), *tet*(K), *erm*(C) (1)	–	–	–
				*mecA*, *blaZ*, *tet*(M), *tet*(K), *msr*(A) (1)	–	–	–
			FOX, PEN, TET, ERI, CLI, GEN,	*mecA*, *tet*(M), *tet*(K), *erm*(B),	–	–	–
			TOB, CIP (2)	*aac(6’)-Ie-aph(2”)-Ia* (2)			
			FOX, PEN, TET (1)	*mecA*, *blaZ*, *tet*(M), *tet*(K)	–	–	–
			FOX, PEN, TET, ERI, CIP (1)	*mecA*, *blaZ*, *tet*(M), *tet*(K), *erm*(C)	–	–	–
			FOX, PEN, TET, SXT, CIP (1)	*mecA*, *blaZ*, *tet*(M), *tet*(K), *dfrG*	–	–	–
	t034 (2)	HUAV (2)	FOX, PEN, TET, CLI, SXT (1)	*mecA*, *tet*(M), *dfrA*, *dfrG*	–	–	–
			FOX, PEN, TET, ERI, TOB, CIP (1)	*blaZ*, *tet*(M), *tet*(K), *ant(4’)-la*	-	–	–
	t108 (1)	HUV (1)	FOX, PEN, ERI, CLI, TET, TOB, CIP, SXT (1)	*mecA*, *blaZ*, *erm*(C), *tet*(M), *tet*(K), *tet*(L), *ant(4’)-la, dfrK*	–	–	–
CC1-MRSA *n* = 4	t127 (4)	HUVM (1)	PEN, FOX, TET, ERI, CLI**^c^** (1)	*mecA*, *tet*(K), *erm*(C)	-	+	-
		HUA (1)	PEN, FOX, TET, ERI, CLI (1)	*mecA*, *tet*(K), *erm*(C)	*lukF/S-PV*	+	E
		HULB (1)	PEN, FOX, TET, ERI, CLI, CIP (1)	*mecA*, *tet*(K), *erm*(C)	–	–	–
		HRV (1)	PEN, FOX, TET, ERI, CLI, TOB (1)	*mecA*, *tet*(K), *erm*(C), *msr*(A), *ant(4’)-la*	–	–	–

**^*a*^Code for the hospitals (H.). HUAV, H. Universitario Arnau Vilanova; HSJ, H. San Jorge; HB, H. Barbastro; HUV, H. Universitari de Vic; HUMS, H. universitario Miguel Servet; HULB, H. Universitario Lozano Blesa; HRV, H. Royo Villanova; CHN, Complejo Hospitalario de Navarra; CUN, Clínica Universitaria de Navarra; HUVM, H. Hospital Universitario Virgen Macarena; HUB, H. Universitario de Burgos; HSP, H. San Pedro; HG, H. de Galdakao; HUA, H. Universitario de Álava; HUD, H. Universitario de Donostia; HUGM, H. Universitario Gregorio Marañon; HUMV, H. Universitario Marques Valdecilla.^*b*^Antimicrobials tested: penicillin (PEN), cefoxitin (FOX), tetracycline (TET), erythromycin (ERY), clindamycin (CLI), ciprofloxacin (CIP), trimethoprim/sulfamethoxazole (SXT), vancomycin, teicoplanin, linezolid, fosfomycin, daptomycin, fusidic acid, mupirocin, gentamicin (GEN), tobramycin (TOB), amikacin and chloramphenicol. ^*c*^Inducible resistance.^*d*^I: intermediate resistance.**

Most of CC398-MSSA isolates (except five) contained the *scn* gene, marker of the IEC system, and the IEC types B, C or E were identified (C: 64%; B: 30.5%) (Table 2). Two of the five isolates lacking the *scn* gene corresponded to the MSSA tetracycline susceptible (TET^*S*^) t011 isolates. One of the two CC398-MSSA/t011/*scn*-negative isolates was recovered in a hospital of a VHPD region and the patient was a pig farmer. The other t011 *scn*-negative isolate was from a hospital of a LPD region (no epidemiological data about patient was obtained). Another CC398-MSSA isolate lacking the *scn* gene was TET^R^, ascribed to *spa*-type t034, *eta*-positive and was recovered in a hospital of a VHPD region. The remaining two CC398-MSSA *scn-*negative corresponded to isolates of *spa*-types t571 and t1451. In addition, the *eta* gene was detected in three other isolates: t571 (*n* = 1) and t1451 (*n* = 2), but neither *etb* nor *lukF/S-PV* were found in this study.

Most of the CC398-MSSA isolates carried the *erm*(T) gene (75%), associated in all cases with the erythromycin-clindamycin^inducible^ (ERY-CLI^Ind^) resistance phenotype (except in one t4030 isolate) (Table 2). This phenotypic/genotypic feature was detected in 87.9% of MSSA-CC398 t571/t1451 isolates, but in none of t011, t034, and t899 isolates. All CC398-MSSA isolates showed susceptibility to tetracycline, except two isolates (of *spa*-types t899 and t034, one each) recovered in two hospitals located in a VHPD region.

No statistical significance was detected when the CC398-MSSA-t571/MSSA rate was correlated to the PD of the surrounding regions (*p* = 0.574) or when correlation was analyzed for C398-MSSA [t571 + t1451]/MSSA and PD (*p* = 0.428).

### Genetic Characterization of C398-MRSA Isolates

The 10 CC398-MRSA isolates were typed as t011 (*n* = 7), t034 (*n* = 2), or t108 (*n* = 1). They were all multidrug-resistant, according to Magiorakos et al. (2012) criteria, and resistance to tetracycline was mediated by the *tet*(M) and *tet*(K) genes, while methicillin-resistance was due to the *mecA* gene. They all lacked the *scn* gene or other virulence genes (Table 2).

### Other Genetic Lineages of Interest Among MRSA-TET^R^ Non-CC398

Four of the 25 MRSA-TET^R^ isolates analyzed in this study were typed as t127/CC1; two were *scn*-negative and were recovered from hospitals of VHPD regions. Another strain was IEC type E and PVL-positive. All these isolates were resistant to multiple antibiotics, including TET and ERY-CLI, mediated by the *tet*(K), and *erm*(C) or *msr*(A) genes (Table 2).

Other *spa*-types were detected among our non-CC398/non-CC1 MRSA-TET^R^ isolates: t1084, t1081, t148, t002, t1818, t2000, and t1597; they were mostly resistant to multiple antibiotics and *scn-*positive.

### Global Comparison of CC398 Isolates

Table 3 shows the main characteristics of the isolates of the lineage CC398, both MSSA and MRSA. Thirty-one of the 44 CC398-MSSA isolates (70.5%) showed the characteristics of the human associated (HA) clade, carrying the IEC system (indicative of the presence of the prophage ΦSa3), absence of the *tet*(M) gene and presence of the *erm*(T) gene. Eight additional CC398-MSSA isolates carried the IEC system (human adaptation marker). This means that 39 of the 44 CC398-MSSA isolates (88.6%) carried the genes of the IEC system. On the contrary, all 10 CC398-MRSA isolates presented the characteristics of the LA clade (absence of IEC, presence of *tet*(M) gene and absence of *erm*(T) gene).

**TABLE 3:** Clade markers detected in 54 CC398 *S. aureus* isolates (both MSSA and MRSA) obtained from blood cultures in a Spanish multicenter study.

Strain type	*spa*-type	Phenotype		Genotype			*scn*	IEC^a^ type (*n*° isolates)	Clade markers			Clade^b^
(*n*° isolates)		TET^R^	ERY^R^-CLI^R^	*tet*(M)	*tet*(K)	*erm*(T)			IEC^a^	*tet*(M)	*erm*(T)	
**CC398-MSSA (*n* = 44)**												
14	t571	–	+	–	–	+	+	5 (B), 9 (C)	+	–	+	HA
13	t1451	–	+	–	–	+	+	10 (C), 3 (B)	+	–	+	HA
2	t4030	–	1/2	–	–	+	+	C (2)	+	–	+	HA
1	t7880	–	+	–	–	+	+	C	+	–	+	HA
1	New	–	+	–	–	+	+	B	+	–	+	HA
4	t571	–	–	–	–	–	+	B	+	–	–	HA
1	t7160	–	–	–	–	–	+	E	+	–	–	HA
1	t1255	–	–	–	–	–	+	E	+	–	–	HA
1	New	–	–	–	–	–	+	C	+	–	–	HA
1	t571	–	+	–	–	+	–	–	–	–	+	?
1	t1451	–	+	–	–	+	–	–	–	–	+	?
2	t011	–	–	–	–	–	–	–	–	–	–	LA?
1	t034	–	+	–	+	–	–	–	–	–	–	LA?
1	t899	+	–	+	–	–	+	B	+	+	–	HA–LA
**CC398–MRSA (*n* = 10)**												
7	t011	+	–	+	+	–	–	–	–	+	–	LA
2	t034	+	–	+	1/2	–	–	–	–	+	–	LA
1	t108	+	–	+	+	–	–	–	–	+	–	LA

*^*a*^IEC: immune evasion cluster. ^*b*^HA, human-adapted; LA, livestock associated; HA-LA, Human-adapted-livestock-associated; ? non defined clade.*

## Discussion

*Staphylococcus aureus* is a leading cause of bacteremia in Europe (Bonnet et al., 2018), and according to our results, CC398 is a relevant lineage among *S. aureus* isolates implicated in bloodstream infections in this multicenter study in Spain, representing 5.3% of total *S. aureus* isolates; interestingly, CC398-MSSA predominated with respect to CC398-MRSA isolates (81.5%). CC398-MSSA has emerged as an invasive subpopulation particularly in France, and in other countries, with increasing prevalence reported over the years. The rate of CC398-MSSA with respect to *S. aureus* in this work (4.3%) is similar to the data previously detected by our research group in one Spanish hospital (5.2%) (Mama et al., 2020b); it is slightly higher than results of a multicenter study performed in France a few years ago (2.3%) (Valentin-Domelier et al., 2011) and lower than more recent studies (Bouiller et al., 2016; Bonnet et al., 2018; Sauget et al., 2019).

Our results show that no statistical correlation exists between the prevalence of CC398-MSSA invasive isolates at hospital level in this multicenter study and the PD of surrounding regions (*p* = 0.108), suggesting that CC398-MSSA is a livestock-independent clade. Severe infections caused by CC398-MSSA isolates, mostly acquired in the absence of animal contact were yet reported (Uhlemann et al., 2012; Kashif et al., 2019).

Concerning CC398-MRSA isolates, all were recovered from hospitals located in regions with VHPD. The significant correlation found between CC398-MRSA/MRSA rate and the PD of surrounding regions (*p* < 0.005) support a previous study which demonstrated that increased pig population density in a region leaded to an increase in CC398-MRSA cases among hospitals of surrounding regions (Ceballos et al., 2019); moreover, that referred study shows that CC398-MRSA is much less frequent among invasive infections than among skin and soft-tissue or respiratory infections (Ceballos et al., 2019).

Most of our CC398-MSSA isolates harbored the IEC system (88.6%) which could suggest human adaptation and corroborates the livestock-independent origin. Nevertheless, some exceptions were detected; the absence of IEC system and the presence of a TET^R^-phenotype in two CC398-MSSA isolates with *spa*-types usually livestock-associated (t034 and t899, linked to *tet*(K) and *tet*(M) genes, respectively) allows us to hypothesize about an evolutive stage in the human or animal adaptation. Resistance to ERY-CLI^Ind^, mediated by *erm*(T) alone or combined with other genes is a recurrent characteristic among our CC398-MSSA isolates (75%), already reported in previous studies (Vandendriessche et al., 2011; Bonnet et al., 2018; Mama et al.,2020a,b). Therefore, the phenotype ERY-CLI^Ind^ associated with the presence of the *erm*(T) gene may be a marker for CC398-MSSA detection.

In the case of CC398-MRSA, TET^R^ was demonstrated to be a good phenotypic marker (Benito et al., 2014). However, none of the CC398-MSSA showed resistance to tetracycline (except two t899 and t034 isolates of VHPD regions). These findings suggest the existence of a specific pheno-genotypic marker for CC398-MSSA (ERY-CLI^Ind^ with *erm*(T) gene, IEC-positive) and for CC398-MRSA (TET^R^-*tet*(M), IEC-negative). In our work, t571 and t1451 were the *spa*-types most frequent among CC398-MSSA isolates. Of note, t571 is the *spa*-type most associated with CC398-MSSA bloodstream infections in Europe (Valentin-Domelier et al., 2011; Vandendriessche et al., 2011; Tavares et al., 2014; Bonnet et al., 2018; Mama et al., 2020b) and it is very unusual among CC398-MRSA isolates. The *spa*-type t1451 has been detected among both CC398-MRSA and CC398-MSSA isolates (Ceballos et al., 2019), although it is much less frequent in CC398-MRSA. Most of MRSA-t1451 isolates lacked the *scn* gene (Van Wamel et al., 2006), but all our MSSA-t1451 isolates carried this gene, with one exception. Statistical analysis showed that PD does not influence CC398-MSSA-t571/MSSA rate (*p* = 0.574) or CC398-MSSA (t571 + t1451)/MSSA (*p* = 0.428).

The CC398-MRSA isolates of our study (*n* = 10) belonged to *spa*-types strongly related to livestock (Zarazaga et al., 2018) and associated to LA-CC398-MRSA in Spanish hospitals (t011, t034, and t108) (Lozano et al., 2012; Benito et al., 2014; Ceballos et al., 2019). The absence of *scn* gene in theses isolates pointed to an animal origin, as expected.

Among the MRSA-TET^R^ non-CC398 isolates of our study, two t127/CC1 isolates (out of four) were *scn*-negative and were recovered from hospitals of VHPD regions, pointing to an animal origin, being considered as LA-CC1-MRSA; the presence of IEC system and PVL genes in another t127/CC1 isolate suggest a probably human-adaptation. The CC1 is a community-associated clonal complex detected usually in humans, but the lineage t127/CC1 is also widely spread in livestock (Lozano et al., 2012; Benito et al., 2014; Zarazaga et al., 2018).

## Conclusion

The lineage CC398 was detected among 5.3% of *S. aureus* isolates of blood cultures of this multicenter study, being CC398-MSSA found in most of the cases (81.5%). Important differences were detected between CC398-MSSA and CC398-MRSA isolates suggesting two different clades, mostly livestock-independent and livestock-associated, respectively. CC398-MRSA isolates were exclusively found in hospitals located in VHPD regions (positive correlation to pig-farming density), and in all cases lacked *scn* gene, characteristic of animal origin. On another hand, CC398-MSSA are more frequently detected in blood cultures in the present study, and no statistical correlation was detected with the PD of the region in which hospitals were located. Resistance to ERY-CLI^Ind^ and presence of *erm*(T) gene could be good markers for livestock-independent CC398-MSSA isolates; moreover, contrary to CC398-MRSA, almost all were tetracycline-susceptible. The characteristics of MRSA and MSSA of CC398 should be further investigated to understand better their clinical and epidemiological burden in Spanish regions.

## Data Availability Statement

The raw data supporting the conclusions of this article will be made available by the authors, without undue reservation, to any qualified researcher.

## Ethics Statement

Ethical review and approval was not required for the study on characterization of bacterial isolates in accordance with the local legislation and institutional requirements.

## Author Contributions

CT and CA conceived and designed the study. CA, EC, JMA, LL-C, CS, AL-C, AB-B, PB, MS, and the members of the Spanish Study group of clinical *S. aureus* CC398 designed and participated in the strain recovery, identification, and susceptibility testing. OM, LR-R, and MI-B performed the molecular characterization of isolates and susceptibility testing. SC realized the statistics analysis. CT, CA, MZ, and OM interpreted the results and performed the first writing of the manuscript. All authors reviewed and approved the manuscript.

## Conflict of Interest

The authors declare that the research was conducted in the absence of any commercial or financial relationships that could be construed as a potential conflict of interest.
